# Probiotic Properties of Exopolysaccharide-Producing *Lactobacillus* Strains Isolated from Tempoyak

**DOI:** 10.3390/molecules23020398

**Published:** 2018-02-13

**Authors:** Eilaf Suliman Khalil, Mohd Yazid Abd Manap, Shuhaimi Mustafa, Amaal M. Alhelli, Parisa Shokryazdan

**Affiliations:** 1 Department of Food Technology, Faculty of Food Science and Technology, Universiti Putra Malaysia, Serdang 43400 UPM, Selangor, Malaysia; eilafsuliman@gmail.com; 2 Department of Dairy Production, University of Khartoum, Khartoum North 13314, Khartoum, Sudan; 3 Halal Products Research Institute, Universiti Putra Malaysia, Serdang 43400 UPM, Selangor, Malaysia; shuhaimi@upm.edu.my; 4 Department of Microbiology, Faculty of Biotechnology and Biomolecular Science, Universiti Putra Malaysia, Serdang 43400 UPM, Selangor, Malaysia; 5 Department of Food Science, Faculty of Food Science and Technology, Universiti Putra Malaysia, Serdang 43400 UPM, Selangor, Malaysia; amaalalhelli@yahoo.com; 6 Agriculture Biotechnology Research Institute of Iran (ABRII), East and North-East Branch, P.O. Box 91735/844, Mashhad, Iran; parisa_shokryazdan@yahoo.com

**Keywords:** antibacterial activity, *Lactobacillus*, exopolysaccharides, probiotics, tempoyak

## Abstract

Tempoyak is a functional Malaysian food (an acid-fermented condiment) which is produced from the pulp of the durian (*Durio zibethinus*) fruit. The current study aimed to isolate and identify potential exopolysaccharide (EPS)-producing *Lactobacillus* strains from tempoyak for potential use as probiotics. Seven isolates (DUR2, DUR4, DUR5, DUR8, DUR12, DUR18, and DUR20) out of 44 were able to produce EPS, and exhibited resistance to acid and bile salt compared to the reference strains *Lactobacillus rhmnosus* (ATCC53103) and *L. plantarum* (ATCC8014). The seven isolated strains belonged to five different species—*L. plantarum*, *L. fermentum*, *L. crispatus*, *L. reuteri*, and *L. pentosus—*which were identified using API 50 CHL and 16S rRNA gene sequences (Polymerase chain reaction, PCR – based). The seven strains displayed different ability to produce EPS (100–850 mg/L). Isolates exhibited a high survivability to acid (pH 3.0), bile salts (0.3%), and gastrointestinal tract model (<70%). Results showed that the auto-aggregation and cell surface hydrophobicity ranged from 39.98% to 60.09% and 50.80% to 80.53%, respectively, whereas, the highest co-aggregation value (66.44%) was observed by *L. fermentum* (DUR8) with *Pseudomonas aeruginosa*. The isolates showed good inhibitory activity against tested pathogens, high antioxidant activity (32.29% to 73.36%), and good ability to reduce cholesterol (22.55% to 75.15%). Thus, the seven tested strains have value as probiotics.

## 1. Introduction

Functional food is a term that refers to a group of foods having therapeutic, prophylactic, and nutritive values [1]. According to Day et al. [2], functional foods are foods or food ingredients that provide additional physiological benefits besides their nutritional value. Over the past decade, there has been an increasing interest in probiotics and recently, there is a growing body of literature that recognizes the importance of the probiotic products and fermented foods as promising functional foods due to their benefits for gut health, disease prevention, and therapy [3,4,5]. According to the Food and Agriculture Organization (FAO) and World Health Organization (WHO) joint report [6], probiotics are those “live microorganisms which when administered in adequate amounts confer a health benefit on the host”. Traditionally, lactic acid bacteria (LAB) represent the main probiotics used in food product processing as starter cultures, pharmaceuticals, and biological control agents [7]. To date, more than 62 genera of LAB are widely used in commercial products as safe fermentation cultures [8]. These mainly include members of the *Streptococcus*, *Leuconostoc*, *Lactococcus*, *Oenococcus*, *Carnobacterium*, *Lactobacillus*, *Pediococcus*, *Enterococcus*, *Tetragenococcus*, *Vagococcus* and *Weisella* [9]. In particular, the *Lactobacillus* species are one of the most widely used groups of bacteria used as probiotics and their usage as microbial food supplements has obtained the status of Generally Recognized As Safe (GRAS) [8]. Lactobacilli are found in the gastrointestinal tract (GIT) of humans and animals, fermented animal and plant products, and most of the commercially available fermented foods.

As probiotics should have the potential to confer health benefits to the host, probiotic strains must have the ability to withstand the stomach’s low pH and the small intestines’ bile salts and pancreatin. In addition, probiotics should also have desirable antibiotic susceptibility patterns and antagonistic to inhibit the enteric pathogens enzyme [10,11]. Moreover, certain probiotic characteristics are required at the cell surface level to colonize the intestines. These include hydrophobicity, auto-aggregation and co-aggregation [12]. Furthermore, probiotics stains need to have some functional attributes such as antioxidative effects, cholesterol assimilation, and immunomodulatory activities [13]. Antagonistic activity and production of antimicrobial compounds is a very important probiotic characteristic which is needed to inhibit the growth of pathogenic bacteria. The antimicrobial activity of probiotic bacteria is mainly through the production of antimicrobial compounds such as organic acids (i.e., lactic acid, acetic acids, butyric acid and, etc.), hydrogen peroxide, and bacteriocins [14]. Exopolysaccharides (EPS) from LAB are widely used in the food industry as viscosifying, stabilizing, gelling, or emulsifying agents, due to their physical and rheological properties [15]. Moreover, EPS from LAB also have beneficial health which can offer protection against the harsh conditions of the gastrointestinal; EPS may also play a role in biofilm formation. Further EPSs may induce positive physiological responses including cholesterol lowering, reduced formation of pathogenic biofilms modulation of adhesion to epithelial cells [16].

Malaysia has a variety of traditional fermented foods and condiments, which have been long produced from different raw materials such as meat, fish, fruits, vegetables, and cereals. Tempoyak is one of these Malaysian fermented products, which is produced from the pulp of the durian fruit (*Durio zibethinus*). Tempoyak is an acid fermented condiment used with special foods like fish and vegetables which is produced through a spontaneous and uncontrolled fermentation process [17]. According to Leisner, et al. [18], LAB are the predominant microorganisms in tempoyak with *Lactobacillus plantarum* as the predominant identified LAB member. However, other species including *L. fersantum*, *L. corynebacterium*, *L. brevis*, *L. mali*, *L. fermentum*, *L. durianis*, *L. casei*, *L. collinoides*, *L. paracasei* and *L. fructivorans* were also reported in tempoyak [17,18,19,20]. Although a considerable number of well-characterized probiotic strains are available around the world, screening for novel strains with specific properties and technologies is still of great interest to improve the probiotic production in order to meet the increasing demand of the market. Moreover, some of these studies concerning the potential health benefits of probiotics effects tend to be strain specific; thus, the aim of this study was to isolate and identify new *Lactobacillus* strains from tempoyak and characterizing their probiotic and functional properties.

## 2. Results

### 2.1. Identification of the Isolates

**Figure 1:**
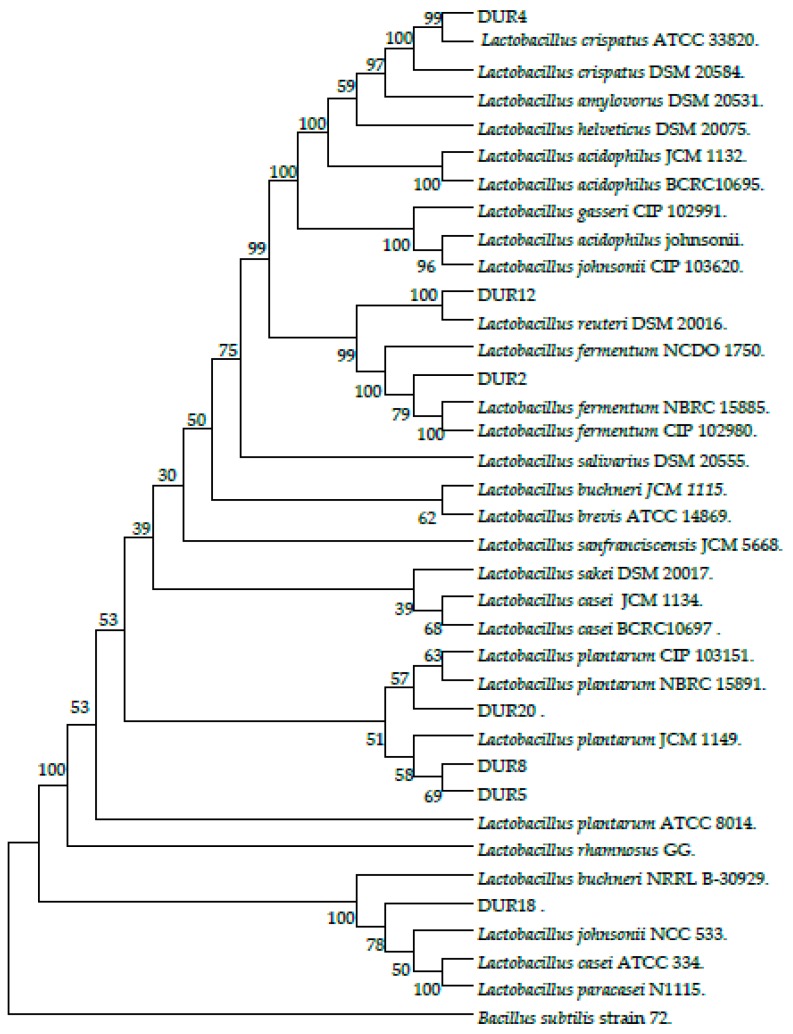
Phylogenetic tree based on the 16S rRNA gene sequences using the neighbor-joining method. The seven isolated strains are (DUR2, 4, 5, 8, 12, 18, and 20). *Bacillus subtilis* was used as an out group nucleotide. Bootstrap values above 50% are indicated at the nodes of the tree. The scale bar represents 0.02-nucleotide substitutes per position.

Forty-four isolates out of 100 showed the typical features of LAB (rod-shaped, Gram-positive, and catalase negative). Seven isolates were chosen for further evaluation of their probiotic characteristics according to a preliminary screening for low pH and bile salt tolerance using optical density (OD) values (data not shown). As presented in Table 1, identification of the isolates using carbohydrates fermentation profile and 16S rRNA gene sequence (99–100% similarity) indicated that the seven strains belonged to five different species of the genus *Lactobacillus*, including one *L. fermentum* (DUR18), three *L. plantarum* (DUR2, DUR5, DUR8), one *L. reutri* (DUR12), one *L. crispatus* (DUR4), and one *L. pentosus* (DUR20). Based on 16S rRNA gene sequences, a phylogenetic relationship between the seven isolates and the reference strains obtained from the GeneBank (29 nucleotides) was constructed to indicate the species and distribution of the selected isolates (Figure 1).

### 2.2. Isolation and Quantitative Determination of Exopolysaccharide

The results showed different ability of the *Lactobacillus* strains to produce EPS (100–850 mg/L) with the highest (850 mg/L) EPS yield recorded by *L. pentosus* (DUR20) followed by *L. reuteri* (DUR12) whereas the lowest yield was produced by strains *L. plantarum* (DUR5) and *L. fermentum* (DUR18) (Table 1). 

### 2.3. Acid and Bile Salts Tolerance

The results for the survival rate of the seven isolated *Lactobacillus* strains along with the two reference strains after exposure to pH 3.0 and 0.3% bile salts for 3 h are shown in Table 2. All strains had the ability to survive under low pH condition with different survival rate among strains. The strain *L. plantarum* (DUR8) had the highest (90.24%) survival rate compared with the reference strain *L. plantarum* ATCC8014 which exhibited the lowest (70.54%) resistance to pH 3.0. Other strains showed survivability to pH 3.0 ranging from 88.10% to 78.68%. All the tested strains showed good ability to withstand the exposure to 0.3% bile salts for 3 h. The highest resistance rate was shown by *L. plantarum* (DUR8, 89.62%), compared with the reference strain *L. plantarum* ATCC8014 (71.31%).

### 2.4. Gastrointestinal Transit Tolerance

Persistance of the isolated *Lactobacillus* strains and the reference strains when exposed to conditions simulating the digestive tract environment are shown in Table 3. In all tested strains, exposure to pepsin at pH 5.0 (G2) had a negligible effect on bacterial count compared to lysozyme exposed strains (G1). However, effects of pH reduction from 5.0 to 3.0 (G3–G5) were varied among the strains as follow: Reduction were observed by *L. fermentum* (DUR18) and *L. plantarum* (DUR8), *L. plantarum* (DUR2), and *L. reuteri* (DUR12) compared with *L. plantarum* ATCC8014 whereas slight increase reported by *L. plantarum* (DUR5) and *L. rhmnousus* ATCC53103. For all strains, decreasing pH to 2.1 (G6) and 1.8 (G7) resulted in a sharp reduction in bacterial count. On the other hand, no significant differences in the bacterial count were found between the intestinal-stressed samples (pH 8.0 with 0.30% bile salts and 0.1% pancreatin, Gi3–Gi5) and the gastric-stressed samples (G3–G5). Except for *L. pentosus* (DUR20), this presented a light increase in the viable cell count compared with the strains *L. plantarum* (DUR5), *L. fermentum* (DUR18), and *L. plantarum* (DUR8).

### 2.5. Bacterial Cell Surface Properties

#### 2.5.1. Auto-Aggregation

The auto-aggregate abilities of the strains are shown in Table 4. All the tested strains exhibited high ability to auto-aggregate with themselves after being incubated at 37 °C for 5 h. The highest auto-aggregation ability was shown by *L. crispatus* (DUR4) and the least auto-aggregation ability was shown by *L. pentosus* (DUR20), while other tested strains exhibited auto-aggregation percentages of more than 40%. 

#### 2.5.2. Co-Aggregation

The co-aggregation results of the *Lactobacillus* strains with five pathogenic bacteria after 4 h of incubation are presented in Table 4. All tested strains indicated some co-aggregation attributes against the pathogens. Among the pathogenic bacteria, the *Lactobacillus* strains showed high ability to co-aggregate with *P. aeruginosa* was 66.44% as the highest level of co-aggregation which was observed for the *L. fermentum* (DUR8). The high co-aggregation ability with *Salmonella* Typhimurium, *E. coli*, methicillin resistant *staphylococcus aureus* (MRSA) ATCC 6538 and *S. aureus* were presented by *L. plantarum* (DUR4), *L. pentosus* (DUR20), *L. reuteri* (DUR12) and *L. crispatus* (DUR4), respectively.

#### 2.5.3. Hydrophobicity

The hydrophobicity assay results are shown in Table 4. The hydrophobicity for the tested strains was highly variable, between 30.8 (for *L. plantarum*, DUR8) and 80.5% (for *L. crispatus*, DUR4). The strains *L. plantarum* (DUR8), *L. pentosus* (DUR20), and *L. plantarum* (DUR5) showed hydrophobicities above 50%, whereas, *L. plantarum* (DUR2), and *L. reuteri* (DUR12) had the lowest percentage of hydrophobicity, with values of 30.8% and 33.0%, respectively, compared with 36.2% exhibited by the reference strain *L. plantarum* ATCC8014.

### 2.6. Antibiotic Susceptibility

The results of antibiotic susceptibility of the *Lactobacillus* strains against tested antibiotics are summarized in Table 5. The result revealed that except for DUR18, DUR5, and DUR4, all other strains were found to be sensitive to AMP, MTZ, CL, and CN antibiotics. In contrast, all strains were resistant to VA except for DUR20.

### 2.7. Bile Salt Deconjugation Activity

The results showed that none of the tested strain displayed BSH activity (data not shown).

### 2.8. Functional Properties

#### 2.8.1. Antagonistic Activity of *Lactobacillus* Strains against Some Pathogenic Strains

Table 6 shows the inhibitory effect of the strains against six enteric pathogenic bacteria. The results showed that all the seven *Lactobacillus* strains and the two references strains were found to have inhibitory activities against the tested enteric pathogens with some variations between the strains (inhibition zone from 3.0 to 16.8 mm). The strain *L. plantarum* (DUR2) had high inhibitory activity towards *Staphylococcus aureus*. Strains *L. plantarum* (DUR5), *L. fermentum* (DUR18), and *L. pentosus* (DUR20) as well as the reference strains, *L. rhamnosus* ATCC 53103 and *L. plantarum* ATCC8014, were found to have a moderate inhibitory effect against the tested pathogens. However, the strain *L. pentosus* (DUR20) showed very low inhibition rate towards *Listeria monocytogenes*. 

The *Lactobacillus* strains with antagonistic activity against the above mentioned pathogens were further assayed for the production of inhibitory materials (bacteriocins, H_2_O_2_, or organic acids) using *E. coli* (ATCC) as an indicators pathogen. Compared to untreated supernatants, neither trypsin nor catalase treatment affected inhibitory activity of the *Lactobacillus* strains against *E. coli*, thus for the tested strains production of bacteriocin nor H_2_O_2_ is not the inhibitory mechanism against the indicator (Table 7). However, inhibitory activity towards *E. coli* was faded or significantly receded (*L. plantarum* DUR5 and *L. rhmnosus* ATCC53103) in pH neutralized supernatants, which suggests the production of organic acids as the main inhibitory mechanism for the tested *Lactobacillus* strains.

Organic acid profiles of the *Lactobacillus* strains are shown in Table 8. Generally, the seven isolated strains showed the capability to produce different organic acid including lactic, acetic, propionic, butyric, caproic, and isobutyric acid. In all strains, non-volatile fatty acid lactic acid was the predominant produced organic acid. The highest amount of lactic acid in the supernatant was produced by the reference strain ATCC53103 (184.41 mM) whereas; the lowest value was 135.87 mM produced by *L. fermentum* (DUR18), the rest of strains showed moderate ability to produce lactic acid. The results showed that the Acetic acid production was relatively low compared with the highest amount were produced by *L. crispatus* (DUR4) (158.23 mM) and 121.76 as the lowest amount produced by the reference strain ATCC8014. Propionic, butyric, caproic, and Isobutyric acids were produced in much lesser amounts compared to lactic and acetic acids. 

#### 2.8.2. Antioxidative Activity

Results of the antioxidant activities of the tested strains are shown in Table 9. All *Lactobacillus* strains exhibited high antioxidative effects with some variation between the strains. The highest capacity was (73.36%) recorded by *L. plantarum* (DUR8) followed by *L. pentosus* (DUR20, 70.57%), which were significantly higher compared to that of the two reference strains ATCC53103 and ATCC8014 (59.15% and 50.71%, respectively). 

#### 2.8.3. Cholesterol Assimilation

All strains had the ability to reduce the cholesterol level in De Man, Rogosa and Sharpe (MRS) broth in presence and absence of bile salts (Table 9). Compared to other strains, *L. crispatus* (DUR4) showed the highest cholesterol removal values (69.53% and 75.15% with and without bile salts, respectively). The strain *L. plantarum* (DUR2) showed the lowest capacity to assimilate cholesterol (22.25% and 25.99% with or without bile salts, respectively). In general, incorporation of bile salts led to higher capacity for cholesterol reduction.

## 3. Discussion

Screening for potential probiotic strains originating from local fermented foods has gained increasing attention because of their perceived health benefits for humans. In our current study, a total of seven *Lactobacillus* strains were isolated from traditional Malaysian tempoyak and intensively studied to evaluate their potential probiotic, functional, and safety properties. In the present study, consistent with previous studies on tempoyak [17,20], *L. plantarum* was the predominant *Lactobacillus* in acid-fermented vegetables and fruits such as cucumber and cassava [21], thus, isolation of *L. plantarum* strains from tempoyak was expected. However, isolation of *L. crispatus*, *L. reuteri* and *L. pentosus* from tempoyak were achieved for the first time in this study. These results may support the hypothesis that fruit (*durian*) related aspects, such as cultivars, growth stage, location, and season may affect the microorganism’s diversity in tempoyak. Besides, fermentation aspects, such as raw material (quality of the durian pulp), equipment and utensils, and stage of fermentation may also affect the microbial diversity.

The ability of LAB to produce EPS is a common trait to lactic starters/probiotics the isolated strains displayed different abilities to produce EPS these referred to different factors include strains, fermentation conditions such as temperature, time and pH and the growth medium used which include carbon and nitrogen sources [16]. Various studies were found that EPS yield produced by *Lactobacillus* were varied among different strains was around 1 g/L when the culture medium were not optimized [22].

Tolerance to the harsh environment of the gastrointestinal tract is one of the main factors limiting the use of microorganisms as live probiotic agents. Acid and bile salt tolerance are indeed considered as essential properties required for LAB survivability in the gut [13]. The capacity of potential probiotic strains to withstand in low pH environment (i.e., as low as pH 2.0) of the stomach is the first challenge before they could successfully reach and colonize the host’s small intestine. Particularly survival at pH 3.0 is considered optimal acid tolerance for probiotic strains. However, food ingestion has a buffering effect, which raises the pH value to 3.0 [23]. Accordingly, isolates were examined for their ability to tolerate pH 3.0 in this study. Compared to the reference strains, the seven isolated *Lactobacillus* strains showed good resistance to low pH with some differences among the strains. These results are in agreement with those obtained from previous studies and who found that resistance of *Lactobacillus* strains of human or animal origin or fermented food when exposed to pH range from 1.0 to 3.0 with wide range due to strain-specific attitude and the surviving percentages of strains were higher at pH 3.0 [24]. As bile salts may cause several negative impacts on the bacterial cells including disorganization of the cell wall, oxidative stress, DNA damage, protein denaturation, and intracellular acidification [25], it is necessary to evaluate the ability of the strains to resist the bile salts when screening for potentially effective probiotics [26]. Bile salts in the gut range between 1.5 and 2% in the hour after food ingestion and it decreases gradually to around 0.3% [27]. However, Gilliland, et al. [28] found that concentration of bile salt in the human small intestine is 0.3% (*w*/*v*). Hence, a concentration of 0.3% of bile salts was used in our study to test bile salts survivability in our isolates. All the seven strains showed good resistance when exposed to 0.3% bile salts. Our findings match those observed in earlier studies by [13,29] who found all nine isolated *Lactobacillus* strains exhibited good tolerance to 0.3% bile salt. this also confirmed by Mathara, et al. [30] found that strains of the *L. acidophilus* group and *L. fermentum* showed the high bile salt tolerance.

The persistence of tested isolates in the digestive tract from the mouth (G1), passage through the gastric tract (G3, G4, G5, G6, and G7) to the intestinal tract (GI3, GI4, GI5) were examined using a gastrointestinal mimic model. Our isolated strains had a great ability to withstand the gastric harsh environment. Such resistance to acidic environment can be attributed to the acidic fermentation conditions in tempoyak, which found to be between pH 3.8 and 4.6 [18]. However, bacterial viable count sharply decreased when pH was reduced to pH 2 and pH 1 (G6 and G7). These findings are in accord with previous results conducted by Peres, et al. [31] who found that ten strains belonging to *L. plantarum* and *L. paraplantarum* exhibited high potency to colonize the various GIT compartments with difference abilities between the strains according to their sensitivity to gastric and intestinal secretions [31,32]. Moreover, isolates were exposed to conditions simulate the gastrointestinal environment where bile salts and pancreatic enzyme cause another challenge. The isolates generally showed high bacterial counts which confirm their capability to tolerate the harsh condition of the gastrointestinal tract.

Bacterial cell properties are a prerequisite for colonization and long-term persistence of bacteria strains in the gut to ensure health benefits of the probiotic bacteria for the human. Bacterial aggregation between microorganisms of the same strain is known as auto-aggregation [33], while aggregation between genetically divergent strains is known as co-aggregation [26]. Both auto-aggregation and co-aggregation abilities are commonly used for preliminary selection of probiotic bacteria [34]. Based on the results of the current study, the seven isolates had the potential to co-aggregate with the following indicator pathogens: *S.* Typhimurium, *E. coli*, *P. aeruginosa*, *S. aureus* and methicillin-resistant *S. aureus* (MRSA) with wide variation between the strains. These results are in agreement with the results obtained by Collado [35], which showed that bacterial co-aggregation with pathogens is a strain-specific feature, depending on the tested strain, indicator pathogen, and other environment factors, such as incubation time. Auto-aggregation and co-aggregation can be used to determine the ability of the probiotics bacteria to form biofilms that protect the hosts and prevent them from being invading by pathogens [26]. Ferreira et al. [36] stated that *Lactobacillus* strains could form a barrier that prevents colonization by pathogenic bacteria through co-aggregation. Thus our strains may play a pivotal role in helping the gastrointestinal tract to get rid of pathogens. Cell surface hydrophobicity properties is an interaction between the microbial cells and host cells in a certain way and it indicates the potential of the bacteria to adhere to the epithelium the gastrointestinal tract [37,38,39,40]. As it is shown in our results, cell surface hydrophobicity values were quite different among strains. The high hydrophobicity value of 80% that obtained by the isolated strain *L. crispatus* (DUR4) in line with the range of 75–80% that have been previously exhibited by *L. acidophilus* as reported by Kos et al. [41].

Antibiotic resistance phenotype is one of the most important prerequisite criteria related to the safety issues, by which strains can be classified as probiotics. This safety issue is used to make sure that probiotic strains are not resistant to antibiotics because resistant strains which harbor acquired and transferable antibiotic resistance genes can transfer these genes to pathogenic microorganisms [11]. In this study, generally, all the isolated strains showed resistance to vancomycin, ciprofloxacin, nalidixic acid and nitrofurantoin. However, the strains showed susceptibility to gentamicin, ampicillin, metronidazole, cephalexin, polymyxin B, bacitracin, erythromycin and sulphafurazole. Consistent with our results Ren et al. [17] found that majority of *Lactobacillus* species are intrinsically resistance to vancomycin which indicates their safety [42]. Also, Wang [43] reported that lactobacilli had susceptibility toward ampicillin and erythromycin, while Gueimonde et al. [42] reported susceptibility of *Lactobacillus* strains toward ampicillin, gentamicin, and erythromycin. The susceptibility to such antibiotics could be explained by the inhibitors of nucleic acid synthesis, which seem to have a low inhibitory effect on the majority of *Lactobacillus* species.

The ability of the probiotic strains to deconjugate bile salts has been raised to be detrimental for probiotic strain selection; as it could maximize its prospects of survival in the gastrointestinal tract. Nevertheless, LAB with active BSH activity have been claimed to lower cholesterol level via hydrolyze bile salts to amino acids and cholesterol, which compensate those amino acids lost during excretion [44]. However, none of our isolates exhibited positive BSH activity. These results are consistent with those by [31,45] who reported a lack of this activity in *Lactobacillus* isolated from environment where the bile salts are absent. 

The capability of LAB to antagonize the pathogenic bacteria in the human intestine is a crucial character to maintain the gut microflora balanced and to inhibit bacterial growth of the pathogens. The antimicrobial activity could be explained by different mechanisms, such as competition for limited nutrients and epithelial attaching sites, and/or production of some antibacterial metabolites, such as organic acids, hydrogen peroxide and bacteriocins [46]. These inhibitive components are mainly found in extracellular parts rather than intracellular fractions [47]. Our results revealed varying inhibition zones against the tested pathogen, and these differences among strains in the antimicrobial activity are in line with results obtained by Buddington [48] who indicated that this feature is strain-dependent [47]. Out of the tested antibacterial metabolites, we found that antimicrobial activity in this study could be due to organic acid production. These results are in accordance with studies which attributed antagonistic activity of LAB isolated from fermented foods to the production of organic acids [20]. Our results indicated high production of lactic acid and acetic acid by the isolates, which confer low pH, particularly acetic acid which has two to four times more lethal impact on pathogens than lactic acid [49]. Production of organic acids by LAB, especially those short-chain fatty acids (SCFA) (i.e., lactic, acetic, propionic and butyric acids) lower the pH of the medium to almost pH 4.0 rendering unfavorable condition for most of the Gram-positive bacteria like *S. aureus* which requires pH 4.5–9.3 to survive [50,51]. This can be explained by the fact that acidic environment causes dissociation and interruption of the transport process in the pathogenic bacterial cells. This antibacterial capability of *Lactobacillus* strains can be effectively used as a biocontrol to protect foods to be invaded by pathogens during the processing.

The ability of lactobacilli to reduce the cholesterol level is a very important criterion to select a potential probiotic with diverse health benefits effects [52]. Previous studies have confirmed that the consumption of fermented foods supplemented with *Lactobacillus* or *Bifidobacterium* spp. have the ability to lower the blood cholesterol [53]. It has been reported that [54] there is a significant relationship between in vitro and in vivo cholesterol reduction abilities of lactobacilli, and numerous in vitro studies have been conducted to investigate the capability of LAB strain to reduce cholesterol level in culture media model [55,56], but it seems to be difficult to compare the results due to the usage of different strains or variation in the concentration of the cholesterol. Our study showed all that the seven strains showed ability to remove cholesterol from the growth medium in the presence of bile salts (25.99–75.15%) these results were in agreement with those obtained by Kim et al. [57] who tested *L. acidophilus* ATCC 43121 and *L. plantarum* KU071, and showed that they had a significant effect on the reduction of cholesterol in the presence of bile salts. Ren et al. [13] tested the capability of eight strains to deplete cholesterol reported that there are wide variations in cholesterol depletion among their tested strains. The probable mechanisms of cholesterol removal activity include cholesterol assimilation in the presence of bile salts, destabilization and co-precipitation of cholesterol micelles which occurred under acidic condition. For example, Mathara at al. [30] reported that in their experiments cholesterol reduction in broth culture media was due to co-precipitation of deconjugated bile salts with cholesterol and binding it to bacterial cells which excrete out of the cell. As our result showed, higher capacity toward cholesterol assimilation was in the presence of bile salts rather than the absence of bile salt, which is in the same line with finding of Miremadi et al. [56] and this may be explained by the co-precipitation of cholesterol with bile salts, however, the binding property of cholesterol to the cell wall is the most likely mechanisms. The previous studies [54] reported a significant relationship between the in vitro ability of lactobacilli to remove cholesterol and in vivo. In this study our strains had a high ability to assimilate cholesterol in vitro.

The antioxidant activity of LAB is one of the well-established property which have been investigated recently and have a great role in the prevention of diseases such as diabetic, cardiovascular and ulcer of the gastrointestinal tract [58]. Several antioxidative components that are integrated into the human antioxidant defense system are derived from foodstuffs and/or provided by gastrointestinal microbiota when LAB colonize and propagate in the gastrointestinal tract [59]. A previous study has shown that numerous *Lactobacillus* strains have radical-scavenging activity [59,60], our results confirm these finding using 2,2-diphenyl-1-picrylhydrazyl (DPPH) free radical, which is routinely used for the antioxidant assay. In the present study, the isolated *Lactobacillus* strains exhibited significant variation in their antioxidant activities (32.29–73.36%), which confirms that it is a strain-specific characteristic. Some of our isolates, *L. pentosus* (DUR20) and *L. fermentum* (DUR18) exhibited scavenging ability higher than the references strain (ATCC53103 and ATCC8014). These highly significant records may be explained by the production of enzymes or cell surface compounds as it was stated by Wang et al. [61]. 

## 4. Materials and Methods

### 4.1. Bacterial Isolates and Culture Conditions

Three batches of tempoyak samples were purchased from local Malaysian markets and transferred aseptically to the laboratory. The samples were plated on MRS Agar media (de Man, Rogosa Sharpe Oxoid Ltd., Hampshire, UK). The plates were incubated anaerobically using Gas-Pack, Anaerogen (Oxoid Ltd.) in an anaerobic jar at 37 °C for 48 h. Individual colonies were picked up and streaked onto MRS agar, and were subcultured three times for purification [62]. The pure selected isolates were cultured in MRS broth (Oxoid Ltd.) and were stocked at −80 °C with 20% glycerol for further uses. Two *Lactobacillus* strains, namely *L. rhamnosus* GG (ATCC 53103) and *L. plantarum* (ATCC 8014), were obtained from the American Type Culture Collection (ATCC, Manassas, VA, USA) and were used as the references strains. The isolates were preliminarily identified using Gram staining, and catalase test [29]. Following the primary tests, a total of 44 potential LAB out of 100 isolates were selected and screened by rapid preliminary tests for acid (pH 2.0) and 0.3% bile salts resistance using the turbidimetric method. After that, seven isolates with the highest acid and bile salts resistance were chosen for further identification and probiotics characterization. Carbohydrates fermentation profiles of the seven isolates were obtained using API 50 CH strips (Bio-Mérieux, Marcy l’Etoile, France) following the manufacturer’s instructions, and strains were identified using API LAB Plus software version 3.3.2 (Bio-Merieux, Marcy l’Etoile, France). 

### 4.2. Isolation and Quantitative Determination of Exopolysaccharide

EPS were isolated from the tested strains using a modified method [63]. EPS were isolated from cell-free supernatants (CFSs) prepared by centrifugation at 8000× *g* for 15 min at 4 °C, then double volume of ethanol (80% *v*/*v*) were added to the samples to precipitate the proteins. The samples then were centrifuged at 10,000× *g* for 30 min, at 4 °C. The pellets were dissolved in distilled water and dialyzed using dialysis kits against distilled water for 24 h at 4 °C with replacement of two times. The phenol-sulfuric acid assay method was used to determine the concentration of EPS [64] using glucose as a standard.

### 4.3. Genotypic Identification Using 16S rRNA Gene Sequences

The genotypic identification of the isolates was conducted by 16S rRNA gene sequencing technique. For amplification of the 16S rRNA gene, two universal primers, 27F (AGAGTTTGATCMTGGCTCAG) and 1492 R (TACGGYTACCTTGTTACGACTT) were used [65,66]. The PCR conditions were initial denaturation at 94 °C for 4 min, 30 cycles of denaturation at 94 °C for 1 min, annealing at 55 °C for 30 s and at 72 °C for 2 min, and a final extension at 72 °C for 5 min. The PCR products were purified using a Qiaquick Polymerase Chain Reaction (PCR) purification kit (Qiagen, Hilden, Germany). DNA sequence data sets were assembled using the Bioedit sequence alignment editor software, (version 7.0.9.0. Ibis Biosciences, Carlsbad, CA, USA) [67]. Sequence similarity values were identified using the basic local alignment search tool (BLAST) (http://blast.ncbi.nlm.nih.gov/Blast.cgi) of the National Centre for Biotechnology Information (NCBI, Rockville, MD, USA) and the accession numbers were obtained from GeneBank (NCBI, Rockville, MD, USA). Phylogenetic tree for nucleotides sequences was built up based on 16S rRNA gene sequences using Molecular Evolutionary Genetic Analysis software version 6 (MEGA 6; The Biodesign Institute, Tempe, AZ, USA), using the Neighbour-joining (NJ) method [68]. Sequence alignments were arranged by CLUSTAL W. A bootstrap was running out with 1000 replicates of the data.

### 4.4. In Vitro Characterization of the Lactobacillus Isolates as Probiotics

#### 4.4.1. Acid and Bile Tolerance

Acid and bile tolerance tests were evaluated as described by Kaushik et al. [58]. For acid tolerance, 18 h culture of each isolate was inoculated (1%) in MRS broth supplemented with 0.05% l-cysteine adjusted to pH 3.0 and MRS broth containing 0.3% bile salt. About 100 μL were taken after 3 h of incubation at 37 °C, serially diluted and cultured onto MRS agar plates. The plates were incubated at 37 °C anaerobically for 48 h, then viable counting numbers (CFU/mL) were recorded and the survival rate was calculated using the Tulumoglu et al. [69] method. The tests were performed for each strain in triplicate. 

#### 4.4.2. Gastrointestinal Transit Tolerance

An in vitro procedure that imitates the gastrointestinal environment was used to assess the viability of the selected strains [70]. Briefly, two daily fresh sterile electrolyte solutions were prepared: solution A (containing NaCl, 6.2 g/L; KCl, 2.2 g/L; CaCl_2_, 0.22 g/L and NaHCO_3_, 1.2 g/L, *w*/*v*) and solution B (containing NaCl, 5 g/L; KCl, 0.6 g/L and CaCl_2_, 0.3 g/L, *w*/*v*) (all materials purchased from Merck, Darmstadt, Germany). A propagation of 35 mL from each culture was harvested (3500× *g* for 15 min at 10 °C). The harvested cells were resuspended in the same volume of sterile electrolyte A, adjusted to pH 6.2 using 1 M NaHCO; an aliquot of 0.1 mL was withdrawn to serve as a control (G1). Then, 35 mL of this cell suspension was mixed with 5 mL of solution A containing lysozyme (0.01% *w*/*v*, to mimic the saliva environment) (G2). To imitate the gastric environment, the cell suspension was mixed with 3 mL of solution A (pH 5.0) containing 0.3% (*w*/*v*) pepsin, and incubated using a shaking incubator (37 °C at 50 g for 20 min) (G3). Then, the pH of the suspension was gradually reduced to 4.1, 3, 2.1, and 1.8 using 1 M HCL; aliquots (0.1 mL) of the suspension were taken after sequential incubation (37 °C at 50 g for 20 min) (G4–G7). To simulate the intestinal environment, the pH of the samples G3, G4, and G5 was adjusted to 6.5 using 1 M NaHCO_3_. Then, 4 mL of solution B containing 0.45% (*w/v*) bile salts and 0.1% (*w*/*v*) pancreatin, and adjusted to pH 8.0 was mixed with the samples and incubated with shaking at 37 °C and 50 g for 120 min (to mimic the duodenum environment conditions) (Gi3, Gi4, and Gi5) from each digestion step (G3, G4, G5 respectively). The survivability of all collected samples was assessed by plating on MRS agar. Survival percentage was calculated via colony counts (CFU/mL) in G1 through Gi5 steps. Each assay from G1 to Gi5 was performed for each strain in duplicate.

### 4.5. Bacterial Cell Surface Properties

#### 4.5.1. Auto-Aggregation Assay

The assay was conducted according to the previously described method [35] with some minor modifications. Harvested cells were washed twice with phosphate buffer solution phosphate buffered saline (PBS) (pH 7.2). The cells were re-suspended in PBS (McFarland standard 0.5), vortexed thoroughly and incubated at 37 °C for 5 h. Aliquots (1 mL) were taken from each sample at 0 and 5 h, and the absorbance was measured at an optical density (OD) of 660 nm using a spectrophotometer (BioMate™-3, Thermo Scientific, Alpha Numerix, Webster, NY, USA). Auto-aggregation (%) for each sample was conducted in triplicates and was calculated as:

[A_0_ − A_5_/A_0_] × 100

where A_5_ represented the absorbance at 5 h of incubation, and A_0_ represented the absorbance at 0 h of incubation.

#### 4.5.2. Co-Aggregation Assay

Bacterial cell suspensions of the seven selected strains, as well as individual pathogenic bacteria (namely, *E. coli* ATCC O157:H7, *S.* Typhimurium ATCC 13076, methicillin-resistant *S. aureus* MRSA ATCC 6538, *S. aureus* ATCC 29247 and *Pseudomonas aeruginosa* ATCC 27853) were prepared based on the method that has been described in the auto-aggregation assay. An equal volume (1 mL) of the cell suspensions of each isolate and a pathogenic strain were mixed and incubated at 37 °C. The absorbance was measured at 600 nm at 0 and 5 h of incubation. Co-aggregation (%) for each sample was calculated as:

[A_0_ − A_5_/A_0_] × 100

where A_0_ was absorbance of the mixed bacterial suspension at 0 h of incubation, and A_5_ was absorbance of the mixed bacterial suspension after 5 h of incubation at 600 nm. Each assay was performed in triplicates.

#### 4.5.3. Bacterial Hydrophobicity

The test was done as described by Doyle and Rosenberg [71] with some minor modifications. Bacterial cells were harvested and washed twice with PBS, resuspended in 5 mL of PBS, and the OD_580_ nm was determined. A sample of 2 mL cell suspension was added to 2 mL of n-hexadecane, organic phase (Merck, Darmstadt, Germany), and vortexed for 2 min. The phases were allowed to separate for 30 min at room temperature. One milliliter of the water phase was discarded, and the optical density (OD580 nm) was determined. The test was carried out for all of the seven *Lactobacillus* strains, and the average OD value was determined. Hydrophobicity (%) was calculated as:

[(A_0_ − A_1_)/A_0_] × 100

where A_0_ is the initial OD and A_1_ is the final OD. The assay for each strain was done in triplicates.

### 4.6. Antibiotic Susceptibility

Resistance to some selected antibiotics was done using the agar disc diffusion method previously described by D’Aimmo, et al. [72]. The isolates corresponding to a McFarland standard 0.5 were spread onto Mueller Hinton Agar (Oxoid Ltd.) using a sterile cotton swab. The antibiotics discs (Oxoid Ltd.) containing: ampicillin (10 μg/mL), erythromycin (15 μg/mL), gentamicin (10 μg/mL), metronidazole (5 μg/mL), vancomycin (30 μg/mL), clindamycin (10 μg/mL), chloramphenicol (30 μg/mL), tetracycline (30 μg/mL), and streptomycin (10 μg/mL) were chosen according to European Food Safety Authority recommendations [73] then the disks were placed onto Mueller-Hinton agar (MHA) and the plates were incubated anaerobically at 37 °C for 24 h. This experiment was performed in triplicate. Resistance rates were interpreted according to microbial cut-off values (mg/mL). Susceptibility result was expressed as resistant (R), moderately susceptible (MS), or susceptible (S).

### 4.7. Bile Salt Deconjugation

The ability of the isolated strains to deconjugate Bile salt was detected using the de Albuquerque, et al. [74] method. Overnight bacterial culture was spreading on MRS agar supplemented with 0.5% (*w*/*v*) taurodeoxycholic acid (TDCA) in compare with control MRS plates. After incubation anaerobically at 37 for 24 h the presence white precipitate of deoxycholate indicated the deconjugation of TDCA.

### 4.8. Functional Properties

#### 4.8.1. Antibacterial Activity

The seven strains and the two references were screened for their inhibitory activity against some pathogenic strains using agar well diffusion assay [31] with some minor modifications. This test was performed against the following pathogenic bacteria: *E. coli* O157:H7 ATCC 43895, *S. Typhimurium* ATCC 13076, methicillin-resistant *S. aureus* MRSA ATCC 6538, *S. aureus* ATCC 29247, *P. aeruginosa* ATCC 27853 and *L. monocytogenes* ATCC 7644. CFS were harvested by centrifugation (8000× *g* for 20 min at 4 °C) from freshly overnight cultured MRS broth media after heating and filtered using sterilized membrane filters (0.22 μm Millipore, Darmstadt, Germany). The pH of the filtered CFS either neutralized to pH 6.5 or used without pH neutralization (control). Then, malted nutrient agar (Oxoid Ltd.) was seeded with 10^7^–10^8^ CFU/mL suspension of the tested pathogenic bacteria and poured into Petri dishes. Upon solidification, wells with 6 mm in diameter were made, and 40 μL of the CFS was filled into each well. The plates were then incubated for 24 h at 37 °C, and the diameters of the formed zones of inhibition around each well were measured. This experiment was performed in triplicate.

#### 4.8.2. Characterization of Inhibitory Substances

CFS of cultured MRS media were used to test production of inhibitory substances such as organic acids, bacteriocin, and hydrogen peroxide as potential antimicrobial compounds against *E. coli* ATCC O157:H7 as indicator following the method described by Jin et al. [49]. For the organic acid test, neutralized CFS were either supplemented with trypsin (EC 3.4.21.4) (final concentration of 1 mg/mL) for 12 h at 37 °C for bacteriocin detection, or catalase (from bovine liver; Sigma-Aldrich, St. Louis, MO, USA) (final concentration 0.5 mg/mL) for hydrogen peroxide detection. Supernatants without any supplementation were used as a control. Both treated, and untreated supernatants (50 μL) were transferred into agar wells. The inhibitory zones around the wells were measured after the incubation for 24 h at 37 °C. The assay was repeated three times in duplicates.

Concentrations of organic acids in extracellular extracts of the isolated and references *Lactobacillus* strains were determined using high-performance liquid chromatography (HPLC) using Aminex-HPX 87H column (300 × 7.8 mm; Bio-Rad, Hercules, CA, USA). The temperature of the column was maintained at 35 °C. An aliquot (20 μL) of the filtered samples was injected into the HPLC equipped with a UV absorbance detector (Waters^®^2996 Photodiode Array) set at 220 nm. Degassed 0.002 M H_2_SO_4_ was used as the mobile phase at a flow rate of 0.6 mL/min. HPLC-grade lactic acid, acetic acid, butyric acid, isobutyric, and propionic acid (Sigma-Aldrich-Co., St. Louis, MO, USA) were used as standards.

#### 4.8.3. DPPH Free Radical Scavenging Activity

The antioxidant activity (free radical scavenging) of the strains was tested according to the method of Son and Lewis [75]. The CFS were harvested, 200 μL of DPPH (6 mg/100 mL of methanol) was added to equal volume of methanol-containing CFS (10 μL CFS + 190 μL methanol). A mixture of 200 μL of DPPH with 200 μL methanol was used as the control, while methanol was served as the blank. The tubes were thoroughly vortexed, incubated at room temperature for 30 min in the dark and the absorbance was measured at 517 nm using a 96 well plate reader (Power Wave X340, BioTek instruments, Inc., Winooski, VT, USA). The antioxidant ability of the candidates was expressed as Trolox equivalent antioxidant capacity (TEAC) using the standard curve of the Trolox in the form of Trolox/mL sample solution.

#### 4.8.4. Cholesterol Assimilation

Cholesterol assimilation was assessed using a method developed by [24]. Briefly, MRS broth cultured media were supplemented with 0.3% *w*/*v* bile salts (Oxoid Ltd.), and 1% (*w*/*v*) water-soluble cholesterol (Sigma-Aldrich Co., St. Louis, MO, USA), and incubated anaerobically at 37 °C for 24 h. A sterilized broth media was used as the control. The supernatants were collected after centrifugation of bacterial cell at 8000× *g* for 15 min at 4 °C. One hundred microliters of the supernatant were mixed with 300 μL of 95% ethanol and 200 μL of 50% *w*/*v* potassium hydroxide. A 10 min heating at 60 °C water bath was performed after each step. Upon cooling, 500 μL of hexane was added to each tube and mixed thoroughly for phase separation. Then, 100 μL distilled water was added and allowed to stand for 10 min at room temperature. A 300 μL aliquot of hexane layer was transferred to a clean tube and evaporated under nitrogen gas. Freshly prepared *o*-phthalaldehyde (400 μL; concentration, 0.5 mg/mL acetic acid) was added to the tube and incubated at room temperature for 10 min. After that, 200 μL of concentrated Sulphuric acid was added and incubated at room temperature for 10 min. Finally, a 300 μL was loaded into a 96-well microplate, and the absorbance was read against reagent blank using a microplate reader at 550 nm. This experiment was performed in triplicate. Cholesterol reduction was calculated as follows:

Cholesterol assimilation (%) = [A_0_ − A/A_0_] × 100

where A_0_ is the un-inoculated control broth, and A is fermented broth.

### 4.9. Statistical Analysis

Data from each assay were analyzed by one-way analysis of variance (ANOVA) using the Minitab version 16.0 statistical package (Minitab Inc., State College, PA, USA). Comparison among treatment means was performed using Turkey’s test. The significance is considered at *p* < 0.05.

## 5. Conclusions

The current study investigated seven *Lactobacillus* strains isolated from fermented durian fruit (tempoyak) for their potential probiotic characteristics. Findings revealed that the seven *Lactobacillus* strains had great potency to withstand the low pH 3.0, 0.3% bile salts and the in vitro model of gastrointestinal conditions. Moreover, the isolated strains showed varied cell surface properties, including auto-aggregation, hydrophobicity and co-aggregation with some pathogens. Furthermore, all the tested strains were able to antagonize the tested pathogenic bacteria, exhibited antioxidant activity and reduced cholesterol from the media with higher potency than the reference strain. Generally, the seven *Lactobacillus* strains isolated from tempoyak have the potential to be used as promising probiotics with functional merits. Further in vivo studies to determine their health benefits and safety properties should be carried out before they can be used as probiotics in food industries.

## Figures and Tables

**Table 1:** Identification of the seven isolates using API 50 CHL kits and 16S rRNA gene.

Isolate Code	Name	Identification by API CHL Kits	Gene Accession No.	Exopolysaccharides (EPS) mg/L
DUR2	*L. plantarum*	*L. plantarum*	KJ026622.1	200
DUR4	*L. crispatus*	*L. crispatus*	KP090108	580
DUR5	*L. plantarum*	*L. plantarum*	AB617650.1	100
DUR8	*L. plantarum*	*L. plantarum*	KP716629	450
DUR12	*L. reuteri*	*L. fermentum*	EU626022	810
DUR18	*L. fermentum*	*L. fermentum*	KF148954	110
DUR20	*L. pentosus*	*L. pentosus*	KC113202.1	850

**Table 2:** Viability of *Lactobacillus* strains after exposure to pH 3.0 and 0.3% bile salts for 3 h.

Strain	pH			Bile Salts		
	Control (6.5) ^1^	3.0 ^1^	SR%	Control (0%) ^1^	0.3% ^1^	SR%
DUR2	8.35 ± 0.01	6.57 ± 0.02	78.68 ^F^	7.12 ± 0.01	6.24 ± 0.05	87.64 ^B^
DUR4	7.34 ± 0.10	6.38 ± 0.10	86.92 ^CD^	7.78 ± 0.03	6.03 ± 0.02	77.51 ^F^
DUR5	7.44 ± 0.01	6.52 ± 0.05	87.61 ^BC^	7.00 ± 0.01	6.03 ± 0.01	86.14 ^C^
DUR8	7.07 ± 0.10	6.38 ± 0.01	90.24 ^A^	7.13 ± 0.02	6.39 ± 0.04	89.62 ^A^
DUR12	7.48 ± 0.01	6.59 ± 0.06	88.10 ^B^	8.13 ± 0.10	6.13 ± 0.01	75.40 ^H^
DUR18	7.39 ± 0.03	6.37 ± 0.05	86.20 ^D^	7.13 ± 0.03	6.06 ± 0.01	84.99 ^D^
DUR20	7.20 ± 0.02	6.26 ± 0.01	86.94 ^CD^	8.13 ± 0.07	6.21 ± 0.02	76.38 ^G^
ATCC53103 *	8.56 ± 0.09	6.90 ± 0.02	80.61 ^E^	8.12 ± 0.05	6.75 ± 0.03	83.13 ^E^
ATCC8014 *	8.24 ± 0.01	5.94 ± 0.04	70.54 ^G^	8.54 ± 0.04	6.09 ± 0.01	71.31 ^I^

^1^ Values represent means ± standard deviation (SD) of log_10_ colony-forming unit (CFU)/mL. ^A–I^ Values within a column with different superscript letters differ significantly (*p* ≤ 0.05). Equal superscript letters indicate no significant differences according to Tukey’s test. SR, survaivability rate. *, commercial reference strains.

**Table 3:** Viable cell count [Log_10_ (CFU/mL)] of seven *Lactobacillus* strains at different gastric (G) and gastrointestinal (GI) digestion stages in a dynamic in vitro model ^1^.

Digestion Stage	Strain								
	DUR2	DUR4	DUR5	DUR8	DUR12	DUR18	DUR20	ATCC53103 *	ATCC8014 *
G1	9.62 ± 0.01 ^aA^	9.59 ± 0.01 ^aB^	8.56 ± 0.04 ^eAC^	8.56 ± 0.01 ^eA^	9.55 ± 0.01 ^aAB^	9.46 ± 0.01 ^bAB^	8.63 ± 0.01 ^eA^	9.19 ± 0.04 ^cB^	9.03 ± 0.01 ^dAB^
G2	9.62 ± 0.01 ^aA^	9.620 ± 0.01 ^aA^	8.57 ± 0.01 ^dC^	8.60 ± 0.01 ^dA^	9.62 ± 0.02 ^aA^	9.30 ± 0.01 ^bB^	8.59 ± 0.01 ^dA^	9.30 ± 0.01 ^bB^	9.03 ± 0.01 ^cAB^
G3	9.35 ± 0.01 ^bBC^	9.43 ± 0.04 ^bAB^	8.59 ± 0.01 ^dBC^	8.60 ± 0.05 ^dA^	9.53 ± 0.01 ^aB^	9.54 ± 0.01 ^aA^	8.59 ± 0.01 ^dA^	9.37 ± 0.01 ^bAB^	9.15 ± 0.03 ^cA^
G4	9.42 ± 0.01 ^aB^	9.32 ± 0.01 ^bAB^	8.62 ± 0.01 ^dBC^	6.55 ± 0.02 ^gBC^	9.42 ± 0.01 ^aC^	9.42 ± 0.01 ^aBC^	7.61 ± 0.01 ^eB^	9.40 ± 0.01 ^aAB^	9.22 ± 0.01 ^cA^
G5	9.33 ± 0.02 ^aBC^	9.33 ± 0.01 ^bAB^	8.89 ± 0.03 ^abA^	6.54 ± 0.02 ^fC^	9.33 ± 0.01 ^bD^	9.40 ± 0.01 ^abBC^	7.19 ± 0.01 ^eC^	9.42 ± 0.01 ^aA^	9.19 ± 0.01 ^cA^
G6	7.98 ± 0.07 ^aD^	7.25 ± 0.1 ^cdeC^	7.87 ± 0.1 ^abD^	4.43 ± 0.2 ^gD^	6.48 ± 0.06 ^fF^	7.46 ± 0.30 ^bcE^	4.74 ± 0.01 ^gD^	6.71 ± 0.2 ^efC^	6.97 ± 0.03 ^deB^
G7	5.46 ± 0.09 ^bE^	6.34 ± 0.3 ^cD^	4.35 ± 0.01 ^cdE^	3.27 ± 0.04 ^fgE^	2.42 ± 0.08 ^hG^	3.81 ± 0.09 ^deF^	2.93 ± 0.3 ^ghE^	4.75 ± 0.04 ^cD^	3.50 ± 0.04 ^efC^
Gi3	9.33 ± 0.01 ^cdBC^	9.52 ± 0.01 ^aAB^	8.75 ± 0.02 ^eAB^	6.72 ± 0.01 ^hB^	9.26 ± 0.01 ^dDE^	9.37 ± 0.01 ^bcBCD^	7.13 ± 0.02 ^gC^	9.41 ± 0.01 ^bAB^	9.14 ± 0.01 ^eA^
Gi4	9.22 ± 0.02 ^cC^	9.48 ± 0.01 ^aAB^	8.72 ± 0.01 ^eBC^	6.67 ± 0.01 ^gBC^	9.26 ± 0.01 ^gDE^	9.34 ± 0.01 ^bCD^	7.41 ± 0.01 ^fC^	9.37 ± 0.01 ^bAB^	9.05 ± 0.02 ^dAB^
Gi5	9.30 ± 0.01 ^abBC^	9.26 ± 0.00 ^bcB^	8.60 ± 0.03 ^eBC^	6.53 ± 0.01 ^gC^	9.21 ± 0.00 ^cE^	9.30 ± 0.01 ^abD^	7.62 ± 0.01 ^fB^	9.35 ± 0.01 ^aAB^	8.99 ± 0.01 ^dAB^

^1^ Values are Mean ± SD of cell counts (CFU/mL) of the *Lactobacillus* strains at various digestion stages (G1 to Gi5) G1, untreated control pH 5.0; G2, pH 4.1; G3, pH 3.0; G4, pH 2.1; G5, pH 1.8; (Gi3:GI4:GI5) pH 6.5 treated with bile salt and pancreatin enzyme at pH 8 from G3, G4 and G5. ^A–G^ Capital letters differences between the digestion steps for each strain, ^a–h^ small letters differences between *Lactobacillus* strains. Equal superscript letters indicate no significant differences according to Tukey’s test. * commercial reference strain.

**Table 4:** Auto-aggregation, co-aggregation ability of the *Lactobacillus* strains with five pathogenic bacteria, and hydrophobicity ^1^.

Strain	Auto-Aggregation	*Salmonella* Typhimurium	*E. coli*	*Pseudomons aeruginosa*	Methicillin resistant *Staphylococcus aureus* (MRSA)	*S. aureus*	Hydrophobicity
DUR2	52.24 ± 0.79	28.18 ± 0.050 ^ABCb^	32.93 ± 0.32 ^ABb^	54.50 ± 0.05 ^ABa^	45.91 ± 0.85 ^ABa^	28.51 ± 0.25 ^BCDb^	35.17 ± 0.92
DUR4	60.09 ± 0.80	27.39 ± 0.80 ^ABCc^	34.15 ± 0.12 ^ABbc^	55.46 ± 0.12 ^ABa^	46.47 ± 0.14 ^Aab^	34.47 ± 0.15 ^ABbc^	80.53 ± 0.98
DUR5	48.10 ± 0.90	29.65 ± 0.60 ^ABCc^	32.85 ± 0.53 ^ABc^	62.82 ± 0.86 ^Aaa^	44.90 ± 0.59 ^ABb^	23.60 ± 0.54 ^ABCc^	54.20 ± 0.70
DUR8	49.88 ± 0.69	30.43 ± 0.06 ^ABd^	33.14 ± 0.17 ^ABc^	66.44 ± 0.90 ^Aa^	44.470 ± 0.91 ^ABb^	32.72 ± 0.95 ^ABCcd^	30.80 ± 0.61
DUR12	47.76 ± 0.69	24.48 ± 0.55 ^Cd^	31.09 ± 0.82 ^Bc^	62.14 ± 0.52 ^ABa^	49.97 ± 0.91 ^Ab^	25.21 ± 0.90 ^Dd^	33.02 ± 0.95
DUR18	47.41 ± 0.80	28.22 ± 0.14 ^ABCe^	30.70 ± 0.29 ^Bc^	61.14 ± 0.49 ^ABa^	36.31 ± 0.10 ^CDb^	24.95 ± 0.05 ^Dd^	56.67 ± 0.24
DUR20	39.98 ± 0.90	29.59 ± 0.19 ^ABCe^	37.42 ± 0.91 ^Ac^	59.81 ± 1.61 ^ABa^	44.15 ± 0.87 ^ABb^	34.67 ± 0.97 ^Ad^	61.23 ± 0.53
ATCC53103 *	40.55 ± 0.85	33.62 ± 0.30 ^Ab^	34.11 ± 0.20 ^ABb^	62.68 ± 0.50 ^Aa^	32.28 ± 0.28 ^Dc^	22.10 ± 0.41 ^Dd^	65.35 ± 0.18
ATCC8014 *	45.80 ± 0.35	29.16 ± 0.30 ^ABCc^	22.94 ± 0.16 ^Ce^	50.05 ± 0.050 ^Ba^	40.20 ± 0.26 ^BCb^	26.60 ± 0.65 ^CDd^	36.15 ± 0.82

^1^ Values are means ± SD. ^A–D^ Capital letters differences between strains per pathogen, ^a–f^ small letters differences between pathogens per strain. Equal superscript letters indicate no significant differences according to Tukey’s test. *, commercial reference strains.

**Table 5:** Antibiotic susceptibility of seven isolated strains of *Lactobacillus* and the two reference strains.

Strain	AMP	E	CN	MTZ	VA	TET	CAM
DUR2	S	S	S	S	R	R	S
DUR4	S	S	S	S	R	R	S
DUR5	S	R	S	S	R	S	S
DUR8	S	S	S	S	R	R	S
DUR12	S	S	S	S	R	S	S
DUR18	S	S	S	S	R	S	S
DUR20	S	S	S	S	R	S	S
ATCC53103 *	S	S	S	S	R	S	S
ATCC8014 *	S	S	S	S	R	S	S

AMP, Ampicillin; E, Erythromycin; GN, Gentamicin; MTZ, Metronidazole; VA, Vancomycin; CAM Chloramphenicol; TET, and Tetracycline. S, Sensitive; R, Resistant. * Commercial reference strains.

**Table 6:** Antibacterial effects of *Lactobacillus* using agar well diffusion assay against 6 pathogens.

Strain	*Salmonella* Typhimurium	*E. coli*	*Pseudomons aeruginosa*	MRSA *	*Staphylococcus aureus*	*Listeria monocytogenes*
DUR2	+	+	+	++	+++	++
DUR4	+	++	++	+	++	++
DUR5	++	++	++	++	++	++
DUR8	++	++	++	++	++	++
DUR12	+	++	+	++	+	++
DUR18	+	++	++	++	++	±
DUR20	++	++	++	++	++	++
ATCC53103 *	++	++	+	+	++	+
ATCC8014 *	+	++	++	++	++	+

Diameter of the inhibition zone: ±, 0–4 mm; +, 4–8 mm; ++, 8–12 mm; +++, >12 mm. *, Commercial reference strains. MRSA, Methicillin Resistant *Staphylococcus aureus*.

**Table 7:** Inhibitory activity of untreated and treated (trypsin, catalase, or adjusted to pH 6.5) *Lactobacillus* supernatants against *E. coli* ^1^.

Strain	Untreated Control	Neutralize pH 6.5 (Organic Acid)	Treated with Trypsin (Bacteriocin)	Treated with Catalase (Hydrogen Peroxide)
DUR2	15.7 ± 0.17	ND	14.7 ± 0.25	15.1 ± 0.94
DUR4	14.2 ± 0.30	ND	14.8 ± 0.18	14.5 ± 0.18
DUR5	13.0 ± 0.04	3.2 ± 0.20	13.5 ± 0.09	13.4 ± 0.50
DUR8	9.7 ± 0.31	ND	9.70 ± 0.50	9.5 ± 0.090
DUR12	12.5 ± 0.09	ND	12.2 ± 0.30	12.0 ± 0.14
DUR18	14.7 ± 0.01	ND	14.0 ± 0.20	14.6 ± 0.11
DUR20	12.8 ± 0.14	ND	12.8 ± 0.19	12.3 ± 0.45
ATCC53103 *	13.5 ± 0.20	6.0 ± 0.82	13.3 ± 0.10	13.6 ± 0.32
ATCC8014 *	10.0 ± 0.16	ND	11.0 ± 0.22	10.7 ± 0.19

^1^ Values are means ± SD. ND, Inhibition zone not detected. *, commercial reference strains.

**Table 8:** Organic acid production (mM) profile of the *Lactobacillus* strains ^1^.

Strain	Lactic Acid	Acetic Acid	Butyric Acid	Isobutyric Acid	Caproic Acid	Propionic Acid
DUR2	ND	ND	ND	ND	ND	ND
DUR4	166.44 ± 0.56 ^b^	158.23 ± 0.54 ^a^	20.65 ± 0.078 ^d^	2.75 ± 0.07 ^d^	4.82 ± 0.02 ^a^	27.86 ± 0.01 ^c^
DUR5	147.82 ± 0.84 ^c^	143.91 ± 0.84 ^b^	19.38 ± 0.07 ^e^	2.69 ± 0.07 ^d^	4.49 ± 0.02 ^b^	26.60 ± 0.17 ^d^
DUR8	156.89 ± 0.38 ^e^	148.65 ± 0.46 ^b^	18.62 ± 0.10 ^f^	2.45 ± 0.15 ^e^	3.89 ± 0.01 ^e^	27.24 ± 0.04 ^c^
DUR12	146.84 ± 0.01 ^c^	140.11 ± 0.07 ^c^	21.94 ± 0.02 ^b^	4.91 ± 0.03 ^c^	3.62 ± 0.01 ^c^	30.67 ± 0.08 ^a^
DUR18	135.87 ± 0.38 ^d^	129.14 ± 0.47 ^d^	16.23 ± 0.08 ^g^	2.74 ± 0.04 ^d^	3.11 ± 0.01 ^d^	24.84 ± 0.01 ^f^
DUR20	146.57 ± 0.01 ^c^	138.92 ± 0.06 ^c^	21.24 ± 0.05 ^d^	4.70 ± 0.05 ^c^	3.28 ± 0.05 ^d^	30.09 ± 0.11 ^b^
ATCC53103 *	184.41 ± 0.01 ^a^	130.102 ± 0.04 ^e^	30.30 ± 0.08 ^a^	5.64 ± 0.02 ^b^	3.74 ± 0.02 ^c^	27.83 ± 0.04 ^c^
ATCC8014 *	164.22 ± 0.02 ^b^	121.760 ± 0.02 ^e^	22.92 ± 0.04 ^b^	6.68 ± 0.05 ^a^	4.36 ± 0.10 ^b^	25.57 ± 0.01 ^e^

^1^ Values are means ± SD. ^a–g^ Small letters differences between strains. Equal superscript letters indicate no significant differences according to Tukey’s test. *, commercial reference strains.

**Table 9:** Antioxidant activity and cholesterol assimilation % of the *Lactobacillus* strains ^1^.

Strain	DPPH %	Cholesterol Assimilation %	
		Without Bile Salt	With 0.3% Bile Salts
DUR2	44.19 ± 0.99 ^g^	22.55 ± 0.71 ^e^	25.99 ± 0.83 ^h^
DUR4	32.29 ± 0.33 ^i^	69.53 ± 1.33 ^a^	75.15 ± 0.26 ^a^
DUR5	54.46 ± 0.70 ^e^	58.29 ± 3.22 ^b^	60.89 ± 1.17 ^c^
DUR8	73.36 ± 0.72 ^a^	26.69 ± 3.19 ^e^	30.37 ± 0.17 ^g^
DUR12	40.28 ± 0.32 ^h^	34.23 ± 1.56 ^d^	45.73 ± 0.30 ^e^
DU18	70.57 ± 0.60 ^b^	67.71 ± 0.87 ^a^	71.38 ± 0.18 ^b^
DUR20	61.304 ± 1.10 ^c^	50.04 ± 0.77 ^c^	56.48 ± 0.16 ^d^
ATCC53103 *	59.15 ± 0.70 ^d^	60.03 ± 1.57 ^b^	61.41 ± 1.35 ^c^
ATCC8014 *	50.71 ± 0.72 ^f^	37.11 ± 0.22 ^d^	40.62 ± 0.40 ^f^

^1^ Values are means ± SD. ^a–i^ Small letters differences between strains. Equal superscript letters indicate no significant differences according to Tukey’s test. *, commercial reference strains. DDPH: 2,2-diphenyl-1-picrylhydrazyl.
